# Revised Transcriptome-Based Gene Annotation for Aspergillus flavus Strain NRRL 3357

**DOI:** 10.1128/MRA.01155-20

**Published:** 2020-12-03

**Authors:** E. Anne Hatmaker, Xiaofan Zhou, Matthew E. Mead, Heungyun Moon, Jae-Hyuk Yu, Antonis Rokas

**Affiliations:** a Department of Biological Sciences, Vanderbilt University, Nashville, Tennessee, USA; b Guangdong Province Key Laboratory of Microbial Signals and Disease Control, Integrative Microbiology Research Centre, South China Agricultural University, Guangzhou, China; c Department of Bacteriology, University of Wisconsin—Madison, Madison, Wisconsin, USA; d Department of Systems Biotechnology, Konkuk University, Seoul, Republic of Korea; e Department of Biomedical Informatics, Vanderbilt University School of Medicine, Nashville, Tennessee, USA; University of California, Riverside

## Abstract

Aspergillus flavus is an agriculturally and medically important filamentous fungus that produces mycotoxins, including aflatoxins, which are potent carcinogens. Here, we generated short- and long-read transcript sequence data from the growth of A. flavus strain NRRL 3357 under both typical and stress conditions to produce a new annotation of its genome.

## ANNOUNCEMENT

Aspergillus flavus (order Eurotiomycetes, phylum Ascomycota) is an agriculturally (1) and medically relevant (2) filamentous fungus that produces diverse secondary metabolites (3). Originally isolated from infected peanut cotyledons in the United States, A. flavus strain NRRL 3357 is available from the ATCC (https://www.atcc.org/). To facilitate the study of the biosynthetic gene clusters involved in secondary metabolism and of the genetic determinants of pathogenicity, we generated a new gene annotation based on transcriptomic sequence data obtained from the growth of strain NRRL 3357 under diverse conditions.

Total RNA was isolated from conidia (asexual spores), mycelia (vegetative cells), and cells undergoing asexual development. Conidia were grown at 30°C on solid glucose minimal medium (GMM) for 2 days prior to RNA extraction. Mycelia were grown at 30°C in liquid GMM, with shaking at 220 rpm for 24 h prior to RNA extraction. Cells undergoing asexual development were grown for 18 h in liquid GMM with shaking at 220 rpm, switched to solid GMM for asexual developmental induction, and grown for an additional 24 h at 30°C. To capture mRNA not produced under typical growth conditions, we also isolated RNA from mycelia grown under three separate stress conditions, salinity (0.6 M NaCl), oxidative stress (5 mM H_2_O_2_), and heat (37°C). After 24 h of normal growth, mycelia were stressed for an additional 24 h in GMM at 30°C (or for 24 h at 37°C for heat stress). All samples were freeze-dried and ground using a mortar and pestle and then chilled with liquid N_2_ to prevent RNA degradation. RNA was isolated from the samples using the Qiagen RNeasy plant minikit and stored at −80°C.

All six samples (RNA from conidia, mycelia, cells undergoing asexual development, and mycelia grown under the three stress conditions) were mixed in equal molar amounts into a single sample for sequencing. We also sequenced the individual samples for mycelia (grown at 30°C in liquid GMM) and heat-stressed mycelia (grown at 37°C in liquid GMM). The Illumina libraries for the three samples were generated using the Lexogen mRNA Sense v2 library preparation kit (Illumina) and were sequenced on the HiSeq X Ten platform (2 × 150-bp paired-end format) by Novogene, Ltd. (Beijing). Totals of 27,714,710 (library from mix of six samples), 29,581,787 (mycelia grown at 30°C), and 24,719,957 (mycelia grown at 37°C) raw reads were generated. An additional Iso-Seq library was prepared from the mix of the six samples, size selected for fragments of ≥4 kb using the BluePippin size selection system (Sage Science), and analyzed on the RS II instrument (Pacific Biosciences), resulting in 5,173,949 reads with a mean length of 2,785 bp. Short and long reads were used together to generate a *de novo* transcriptome assembly using Trinity v.2.10.0 (4). As part of the Trinity assembly pipeline, low-quality bases and adaptor contamination were trimmed from the Illumina reads using Trimmomatic v.0.39.0 (5). Short reads were also used for genome-guided assembly using Trinity.

Initial gene models were generated using default parameters for MAKER v.2.31.9 (6), which included three *ab initio* gene predictors, AUGUSTUS v.3.3.3 (7), GeneMark-ES v.4.39 (8), and SNAP v.2013-11-29 (9). Exonerate v.2.4.0 (10) was provided for alignment to the MAKER pipeline. Isoforms were added to PASA v.2.3.3 (11) from both the *de novo* and genome-guided transcriptome assemblies as “EST evidence.” The new annotation predicts 14,313 genes and 14,699 proteins. We compared our annotation to the Aspergillus flavus NRRL 3357 genome annotations from AspGD and NCBI (accession number GCA_000006275.2) using BUSCO v.3.0.2 (12), which estimated a copy number of 4,046 preselected genes considered single copy within the OrthoDB (13) database (Table 1). The new annotation contains 96.3% of these 4,046 genes, substantially higher than the percentages contained by the previous two annotations (AspGD, 90.5%; NCBI, 88.0%; Table 1). The transcriptome-based annotation contains 12,569 genes which overlap one or more gene models in the previous NCBI annotation, as well as 1,744 predicted genes without any overlap with the previous annotation, suggesting that our transcriptome-based annotation provides new information for studying A. flavus.

**TABLE 1:** Summary of BUSCO assessments of the Aspergillus flavus NRRL 3357 genome assembly annotations

Type of data	No. (%) of genes determined by BUSCO		
	Complete	Fragmented	Missing
Annotation (NCBI)^*a*^	3,559 (88.0)	400 (9.9)	87 (2.1)
Annotation (AspGD)^*b*^	3,659 (90.5)	261 (6.5)	126 (3.0)
Annotation (this study)	3,896 (96.3)	106 (2.6)	44 (1.1)

a See ftp://ftp.ncbi.nlm.nih.gov/genomes/all/GCF/000/006/275/GCF_000006275.2_JCVI-afl1-v2.0/GCF_000006275.2_JCVI-afl1-v2.0_protein.faa.gz.

b See http://www.aspergillusgenome.org/download/sequence/A_flavus_NRRL_3357/current/A_flavus_NRRL_3357_orf_trans_all.fasta.gz.

### Data availability.

A. flavus NRRL 3357 RNA sequences from this study have been deposited under Sequence Read Archive (SRA) study SRP213830, with individual samples deposited in the SRA under accession numbers SRX6419472, SRX6419473, SRX6419474, and SRX6419475 and BioSample accession numbers SAMN12235769 through SAMN12235771. This whole-genome sequencing project has been deposited at DDBJ/ENA/GenBank under the accession number AAIH00000000. The annotation has been updated for A. flavus NRRL 3557 assembly accession number GCA_000006275.3 (GenBank), and the annotation described in this paper is the third version, AAIH03000000.
